# Association Between Cardio-Ankle Vascular Index and Heart Failure Outcomes: Insights From a Prospective Multicenter Cohort

**DOI:** 10.1016/j.jacadv.2025.102187

**Published:** 2025-08-29

**Authors:** Toru Miyoshi, Kohji Shirai, Shigeo Horinaka, Jitsuo Higaki, Shigeo Yamamura, Atsuhito Saiki, Mao Takahashi, Takafumi Okura, Kazuhiko Kotani, Takuro Kubozono, Ryo Yoshioka, Hajime Kihara, Koji Hasegawa, Noriko Satoh-Asahara, Osamu Yamaguchi, Hiroshi Ito

**Affiliations:** a Department of Cardiovascular Medicine, Okayama University Graduate School of Medicine, Dentistry and Pharmaceutical Sciences, Okayama, Japan; b Department of Internal Medicine, Mihama Hospital, Chiba, Japan; c Department of Cardiovascular Medicine, Dokkyo Medical University, Mibu, Japan; d Department of Cardiology, South Matsuyama Hospital, Matsuyama, Japan; e Faculty of Pharmaceutical Sciences, Josai International University, Chiba, Japan; f Centre of Diabetes, Endocrine and Metabolism, Toho University Sakura Medical Centre, Sakura-City, Japan; g Division of Cardiovascular Medicine (Sakura), Department of Internal Medicine, Faculty of Medicine, Toho University, Sakura-City, Japan; h Department of Cardiology, Saijo Central Hospital, Saijo, Japan; i Division of Community and Family Medicine, Jichi Medical University, Shimotsuke, Japan; j Department of Cardiovascular Medicine and Hypertension, Graduate School of Medical and Dental Sciences, Kagoshima University, Kagoshima, Japan; k Department of Cardiovascular Medicine, Okayama Kyokuto Hospital, Okayama, Japan; l Department of Internal Medicine, Kihara Cardiovascular Clinic, Asahikawa, Japan; m Division of Translational Research, Clinical Research Institute, National Hospital Organization Kyoto Medical Centre, Kyoto, Japan; n Department of Endocrinology, Metabolism, and Hypertension Research, Clinical Research Institute, National Hospital Organization Kyoto Medical Centre, Kyoto, Japan; o Department of Cardiology, Pulmonology, Hypertension & Nephrology, Ehime University Graduate School of Medicine, Toon, Japan; p Department of General Internal Medicine 3, Kawasaki Medical School, Kurashiki, Japan

**Keywords:** arterial stiffness, cardio-ankle vascular index, heart failure

## Abstract

**Background:**

Heart failure (HF) is a substantial public health concern associated with poor prognosis and limited tools for early prediction. Arterial stiffness contributes to the development of HF, particularly with a preserved ejection fraction. The cardio-ankle vascular index (CAVI) is a noninvasive, pressure-independent marker of arterial stiffness. However, its prognostic value in HF remains unclear.

**Objectives:**

We aimed to evaluate whether CAVI independently predicts HF-related hospitalizations in patients with cardiovascular risk factors and whether it adds prognostic value beyond conventional risk factors.

**Methods:**

This subanalysis of the multicenter prospective CAVI-J (Prospective Multicenter Study to Evaluate Usefulness of CAVI in Japan) study included 2,932 Japanese adults aged 40 to 74 years with cardiovascular risk factors. Participants were stratified into CAVI tertiles and followed up for a median of 4.9 years. The primary outcomes were HF-related hospitalizations and cardiovascular death. Cox proportional hazards models were used to assess the risk, and the incremental prognostic value was evaluated using global chi-square statistics and reclassification indices.

**Results:**

Primary outcomes were recorded in 32 patients (1.1%). High CAVI (≥9.0) was independently associated with an increased risk (HR: 3.18; 95% CI: 1.09-9.31; *P* = 0.034). When CAVI was added to a model with known conventional risks, the global chi value increased from 8.82 to 18.77 (*P* = 0.002), and the net reclassification index was 0.621 (*P* < 0.001).

**Conclusions:**

In this exploratory study, elevated CAVI was associated with HF-related hospitalizations and cardiovascular death and modestly improved the risk prediction beyond conventional risk factors. However, the discriminative ability remained limited, and further studies are warranted to establish its clinical utility.

Heart failure (HF) is a global health issue with an increasing prevalence and poor outcomes.^1^ In the United States, over 6 million adults are affected, and this number is projected to exceed 8 million by 2030.^2^ HF has a 5-year mortality rate comparable to that of many cancers and is a leading cause of hospitalization among older adults.^3^ Despite treatment advances, the prognosis remains poor, and hospitalization imposes a significant burden.^2^ A key unmet need is the early identification of individuals at high risk of HF to enable timely preventive interventions. However, conventional risk factors such as hypertension, diabetes, and coronary artery disease are widespread but not specific. Although many patients with risk factors remain unaffected, others develop it despite the risk factor control.^4^ Existing HF risk scores show limited discrimination and are not routinely used in practice.^3^ Better tools are required to identify subclinical cardiovascular changes in vulnerable patients before overt HF occurs.

Arterial stiffness has emerged as a promising marker and mechanistic contributor to HF, particularly HF with preserved ejection fraction (HFpEF).^5^^,^^6^ It increases left ventricular afterload and disrupts pressure-flow dynamics, promoting diastolic dysfunction.^7^, ^8^, ^9^, ^10^ Although pulse wave velocity (PWV) is a conventional stiffness measure, it is pressure-sensitive and technically demanding.^11^ Carotid-femoral PWV measurement requires precise instrumentation and is affected by acute blood pressure changes. The cardio-ankle vascular index (CAVI) is a noninvasive stiffness index that addresses many of these limitations.^12^ CAVI assesses stiffness from the aortic root to the ankle, capturing both the central and peripheral arteries. CAVI values are relatively stable across blood pressure fluctuations, making them pressure-independent indicators of arterial properties.^13^ It can be measured easily using cuff-based systems and is highly reproducible. Elevated CAVI is associated with coronary artery disease, stroke, and kidney disease.^14^ However, its prognostic value in predicting HF remains unclear. Although a few studies suggest that CAVI is higher in patients with HFpEF and may predict HF hospitalization, large multicenter data are lacking.^15^^,^^16^

Thus, we aimed to evaluate whether baseline CAVI predicts a composite endpoint of HF-related hospitalizations and cardiovascular death in a large prospective cohort of individuals with cardiovascular risk factors and to assess whether adding CAVI improves risk prediction beyond traditional factors.

## Methods

### Study design and population

This study was a subanalysis of the CAVI-J (Prospective Multicenter Study to Evaluate Usefulness of CAVI in Japan), a multicenter, prospective cohort study designed to investigate the prognostic utility of CAVI for cardiovascular events in individuals with cardiovascular risk factors (NCT01859897).^17^ The details of the inclusion and exclusion criteria are described in the Supplemental Appendix and protocol paper.^18^ The study enrolled patients aged 40 to 74 years with at least 1 of the following: type 2 diabetes mellitus,^19^ hypertension (categorized as high risk according to the Japanese Society of Hypertension Guidelines for the Management of Hypertension, 2009),^20^ metabolic syndrome,^8^ stage 3 chronic kidney disease,^21^ or a history of coronary artery disease or cerebral infarction. The exclusion criteria included age <40 or >75 years, ankle-brachial index ≤0.9, atrial fibrillation, HF with NYHA functional class III or IV, left ventricular (LV) ejection fraction (LVEF) < 40%, active malignancy, severe renal dysfunction (estimated glomerular filtration rate <30 mL/min/1.73 m^2^ or dialysis), use of systemic steroids or immunosuppressants, liver cirrhosis, or any other condition deemed unsuitable by the attending physician.

Between May 2013 and December 2014, 3,026 patients were recruited from 63 sites across Japan. After excluding individuals with missing follow-up data or those who withdrew consent, 2,932 patients were included in the present analysis. All the participants provided written informed consent. The study protocol was approved by the institutional review boards of all participating centers and complied with the Declaration of Helsinki.

### Measurement of CAVI

Baseline CAVI was measured using a validated device (VaSera; Fukuda Denshi), based on the stiffness parameter β, and calculated using the formula: CAVI = a{(2ρ/ΔP) × ln(Ps/Pd) × PWV^2^} + b, where Ps and Pd are systolic and diastolic blood pressures, respectively; ΔP is Ps − Pd; and ρ is blood density (1.05 g/cm^3^); and a and b are constants.^12^ For the calculation of PWV, the arterial path length (L) from the origin of the aorta to the ankle is required. In the automatic measurement mode of the VaSera device, L is estimated from the subject’s body height using the following equation: L = 7.76885 × body height–17.536.^22^ Measurements were performed in the supine position after a 5-minute rest using electrocardiography electrodes on the wrists, a phonocardiographic microphone on the sternum, and blood pressure cuffs on the arms and ankles. All CAVI data were reviewed at a central office to ensure quality, and remeasurement was requested when the data quality was insufficient.

### Outcome measures

The primary outcome of this subanalysis was a composite of cardiovascular death or HF-related hospitalizations, including both HFpEF and HF with reduced ejection fraction (HFrEF). HFpEF was defined as HF with LVEF ≥50% and HFrEF as HF with LVEF <50%.^1^ Clinical events were assessed annually by investigators at each site and adjudicated by a blinded Clinical Event Review Committee using prespecified definitions (Supplemental Appendix).

### Covariates

Blood pressure was measured twice using an automated sphygmomanometer with the participants in the sitting position after a five-minute rest. The mean of the 2 measurements was used in the present analysis. Serum total cholesterol and high-density lipoprotein cholesterol levels were determined enzymatically. Obesity was defined as a body mass index >30.0 kg/m^2^. All clinical examinations and blood tests were conducted on the same day.

### Statistical analysis

Categorical data were presented as absolute numbers and percentages. Continuous data are presented as mean (SD). Patients were categorized into tertiles based on CAVI values: T1 (≤8.00), T2 (8.05-8.95), and T3 (≥9.00). Kaplan-Meier curves with log-rank tests were constructed to evaluate time-to-event outcomes.

Cox proportional hazards models were used to estimate HRs and 95% CIs after adjusting for covariates, including age and sex. CAVI was analyzed both as a categorical variable (tertiles and receiver operating characteristic [ROC]-derived cut points) and as a continuous variable to evaluate dose–response associations. To evaluate the added prognostic contribution of CAVI, we assessed the improvement in model goodness-of-fit using the Akaike information criterion and global chi-square statistics, as well as changes in model discrimination and reclassification using the C-statistic, the net reclassification index, and integrated discrimination improvement. ROC curve analysis was also performed to assess discrimination and to identify the optimal cutoff value. The baseline model included known predictors, including age (≥65 years), sex, hypertension, diabetes, total cholesterol, history of cardiovascular disease, and stroke.

As a sensitivity analysis, a competing risk analysis using the Fine-Gray regression method was performed, considering cardiovascular death as a competing risk factor for HF-related hospitalization. The Fine-Gray subdistribution hazard model was used to estimate HRs and 95% CIs, adjusting for covariates, including age and sex. A 2-tailed test, *P* < 0.05, was considered significant. Statistical analyses were performed using JMP Pro (version 18, SAS Institute Japan) and EZR (Saitama Medical Center), which is a graphical user interface for R (R Foundation for Statistical Computing).^23^

## Results

### Baseline characteristics

Table 1 shows baseline characteristics of this study population. A total of 2,932 patients were included in the analysis and stratified into 3 groups based on CAVI tertiles: T1 (≤8.00, n = 956), T2 (8.05-8.95, n = 960), and T3 (≥9.00, n = 1,016). The mean age increased significantly across the groups (*P* for trend <0.001), and the proportion of male patients was higher in the elevated CAVI group (*P* < 0.001). The prevalence of hypertension, diabetes mellitus, chronic kidney disease, and a history of coronary artery disease or cerebral infarction increased significantly with increasing CAVI levels (*P* = 0.004, *P* = 0.008, *P* = 0.002, and *P* = 0.040, respectively), whereas obesity was inversely associated with CAVI (*P* < 0.001). There were no significant differences in smoking status, physical activity, or the use of antihypertensive or lipid-lowering medications. However, the use of insulin, oral antidiabetic agents, and antiplatelet agents was significantly more frequent in the highest tertile group (*P* < 0.001 and *P* = 0.016, respectively).

**Table 1:** Baseline Characteristics According to CAVI

	CAVI			*P* Value (Across groups)
	T1 (≤8.00) (n = 956)	T2 (8.05‒8.95) (n = 960)	T3 (≥9.00) (n = 1,016)	
Age, mean (SD), y	58.8 (9.0)	63.8 (7.2)	66.7 (5.5)	<0.001
Male	587 (61.4)	655 (68.2)	759 (74.7)	<0.001
Systolic blood pressure, mean (SD), mm Hg	130.6 (15.6)	132.1 (15.7)	136.6 (17.5)	<0.001
Diastolic blood pressure, mean (SD), mm Hg	80.0 (11.2)	79.5 (11.2)	80.4 (11.9)	0.198
Hypertension	833 (87.1)	837 (87.2)	927 (91.2)	0.004
Hypertension (high-risk)	769 (80.4)	771 (80.3)	791 (87.7)	<0.001
Diabetes mellitus	705 (73.4)	703 (73.2)	801 (78.8)	0.006
Metabolic syndrome	276 (28.9)	275 (28.6)	269 (26.5)	0.422
Chronic kidney disease	336 (35.1)	362 (37.7)	427 (42.0)	0.006
History of coronary artery disease or cerebral infarction	339 (35.5)	370 (38.5)	406 (40.0)	0.111
Total cholesterol, mean (SD), mg/dL	186.8 (34.4)	184.0 (35.7)	180.9 (33.3)	0.001
HDL cholesterol, mean (SD), mg/dL	55.3 (15.1)	55.2 (16.1)	54.3 (14.9)	0.258
Obesity	209 (21.9)	87 (9.1)	63 (6.2)	<0.001
Smoking habits	410 (42.9)	430 (44.8)	470 (46.3)	0.133
Regular Exercise	321 (33.6)	341 (35.5)	364 (35.8)	0.321
Medications				
Antihypertensive agents	743 (77.7)	731 (76.2)	786 (77.4)	0.530
Insulin	41 (4.3)	45 (4.7)	88 (8.7)	<0.001
Antidiabetic agents	295 (30.9)	364 (37.9)	450 (44.3)	<0.001
Lipid-lowering agents	579 (60.6)	600 (62.5)	626 (61.6)	0.684
Antiplatelet agents	327 (34.2)	364 (37.9)	401 (39.5)	0.047

Values are n (%) unless otherwise indicated.

CAVI = cardio-ankle vascular index; HDL = high-density lipoprotein.

### Association between CAVI AND HF events

During a median follow-up of 4.9 years, the primary outcome, a composite of cardiovascular death or HF-related hospitalizations, occurred in 32 patients (1.1%). Of these, 21 were hospitalized for HF-related events (18 with HFpEF, 3 with HFrEF), with 2 having experienced prior myocardial infarction. The median follow-up times in the T1, T2, and T3 groups were 55.7, 54.8, and 54.2 months, respectively, with no statistically significant differences among the groups.

**Figure 1:**
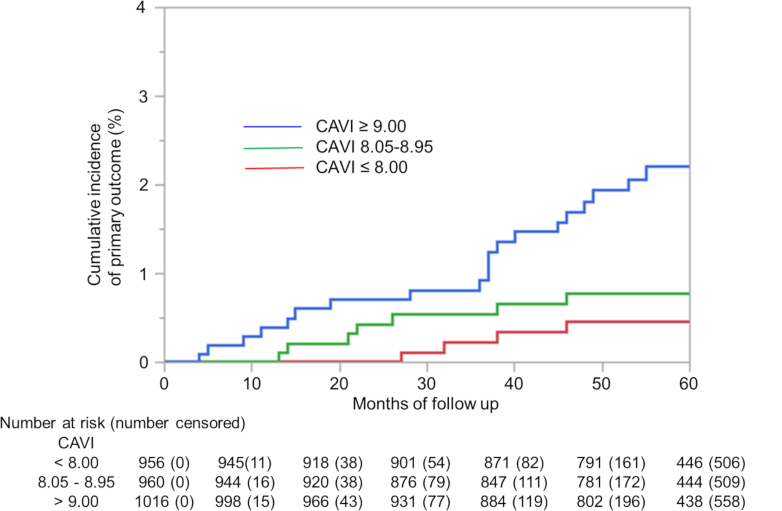
**Cumulative Incidence of the Primary Outcome According to CAVI Tertiles** Kaplan-Meier curves showing cumulative incidence of the primary outcome (composite of heart failure hospitalization or cardiovascular death) stratified by CAVI tertiles: T1 (≤8.0), T2 (8.05-8.95), and T3 (≥9.0). The high CAVI group (T3) exhibited a significantly higher event rate than the lower tertiles (log-rank, *P* < 0.001). The number at risk is indicated on the x-axis. CAVI = cardio-ankle vascular index.

Kaplan-Meier survival analysis showed a significant stepwise increase in the cumulative incidence of the primary outcome across the CAVI groups (log-rank *P* < 0.001) (Figure 1). The multivariable Cox regression analysis, adjusted for age and sex, revealed that patients with CAVI ≥9.00 had a significantly higher risk of the primary outcome (HR: 3.18; 95% CI: 1.09-9.31; *P* = 0.034), whereas the intermediate group (CAVI 8.05-8.95) did not reach statistical significance (HR: 1.26; 95% CI: 0.39-4.11; *P* = 0.697). When modeled as a continuous variable, each 1-point increment in CAVI was independently associated with an increased risk (adjusted HR: 1.59; 95% CI: 1.23-2.06; *P* < 0.001) (Table 2).

**Table 2:** Association Between CAVI and Primary Outcome

CAVI	Univariable			Multivariable^a^		
	HR	95% CI	*P* Value	HR	95% CI	*P* Value
Primary outcome						
<8.0	(reference)			(reference)		
8.0-9.0	1.421	0.451-4.474	0.549	1.26	0.39-4.11	0.697
>9.0	3.878	1.455-10.332	0.007	3.18	1.09-7.31	0.034
CAVI per 1 index	1.65	1.29-2.06	<0.001	1.59	1.23-2.06	<0.001

Abbreviations as in Table 1.

a Adjusted for age and sex.

### Sensitivity analysis

Sensitivity analysis treating cardiovascular death as a competing risk confirmed the robustness of these findings. In the Fine-Gray model, patients with high CAVI (>9.0) had a significantly increased risk of HF hospitalization compared with those with CAVI <8.0 (adjusted HR: 6.44; 95% CI: 1.47-28.38; *P* = 0.013), whereas intermediate CAVI (8.0-9.0) was not significant (adjusted HR: 3.10; 95% CI: 0.68-14.13; *P* = 0.140). When modeled as a continuous variable, each 1-unit increase in CAVI was associated with a 57% higher risk of HF hospitalization (HR: 1.57; 95% CI: 1.16-2.14; *P* = 0.004) (Supplemental Appendix).

### Prognostic discrimination and incremental value of CAVI

ROC curve analysis identified an optimal CAVI cutoff of 8.9 for predicting the primary outcome. The area under the curve was 0.679, with a sensitivity and specificity of 71% and 62%, respectively, indicating modest discriminative ability. To evaluate the incremental prognostic utility, CAVI (>8.90) was added to a baseline risk model comprising age, sex, hypertension, diabetes mellitus, total cholesterol, and history of cardiovascular disease. This resulted in some improvement in model performance: the Akaike information criterion decreased from 357.2 to 349.3, and the global chi-square statistic increased from 8.82 to 18.77 (*P* = 0.002). However, the increase in C-statistic was not significant (from 0.655 to 0.715; *P* = 0.141), and the overall discriminative performance remained below the generally accepted threshold (area under the curve >0.75) for strong clinical utility. Nevertheless, the addition of CAVI significantly improved reclassification and discrimination metrics: the net reclassification index was 0.621 (95% CI: 0.298-0.944; *P* < 0.001), and the integrated discrimination improvement was 0.318 (95% CI: 0.110-0.525; *P* < 0.001) (Table 3).

**Table 3:** Prognostic Value of CAVI for Primary Outcome After Addition to a Model With Risk Factors

	AIC	Global Chi-Square Test Score	C-Statistics	NRI (95% CI)	IDI (95% CI)
Baseline model	357.2	8.820	0.655		
With CAVI (>8.9) added	349.3	18.77	0.715	0.621 (0.298-0.944)	0.318 (0.110-0.525)
		(*P* = 0.002)	(*P* = 0.141)	(*P* < 0.001)	(*P* < 0.001)

The baseline model included age, sex, hypertension, diabetes mellitus, total cholesterol level, and history of cardiovascular disease or stroke.

AIC = Akaike information criterion; IDI = integrated discrimination improvement; NRI = net reclassification index; other abbreviations as in Table 1.

## Discussion

In this prospective cohort study, we found that elevated arterial stiffness, as measured by a CAVI ≥9.0, was significantly associated with an increased risk of HF-related hospitalization and cardiovascular death. This association remained significant after adjusting for conventional risk factors. Kaplan-Meier curves demonstrated a higher cumulative incidence of HF-related hospitalization in the high CAVI group. Although the addition of CAVI to the baseline models resulted in improvements in discrimination and reclassification metrics, the overall discriminative ability remained modest, and the incremental value should be interpreted with caution (Central Illustration).

**Central Illustration:**
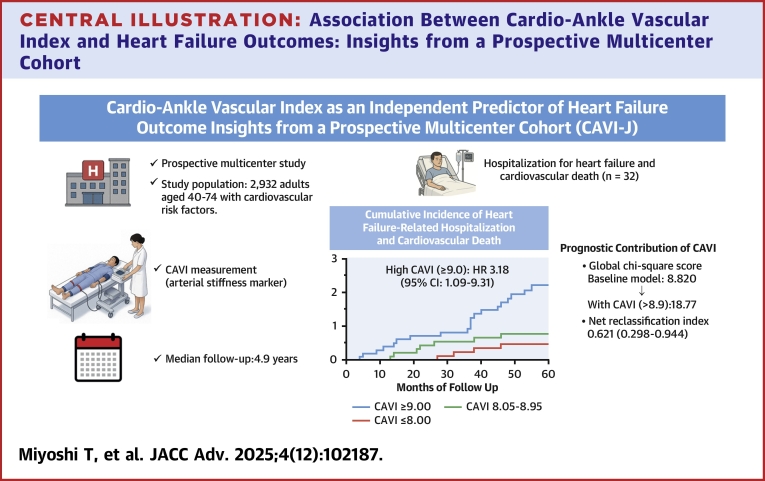
**Association Between Cardio-Ankle Vascular Index and Heart Failure Outcomes: Insights from a Prospective Multicenter Cohort** CAVI = cardio-ankle vascular index; CAVI-J = Prospective Multicenter Study to Evaluate Usefulness of CAVI in Japan.

Although our study reports an association between CAVI and HF-related hospitalizations rather than the composite outcome, these results need to be interpreted cautiously as the numbers in the subgroup are very small. By explicitly considering cardiovascular death as a competing risk, our analysis demonstrated that the prognostic role of CAVI is not simply a reflection of overall cardiovascular mortality but instead specifically identifies individuals at heightened risk of developing overt HF requiring hospitalization. This distinction is important, as hospitalization events directly reflect symptomatic disease progression and impose substantial burdens on patients and health systems. Thus, the competing risk findings underscore the clinical utility of CAVI as a vascular biomarker that captures vulnerability to HF decompensation beyond conventional risk stratification.

The pathophysiological link between arterial stiffness and HF, particularly HFpEF, is supported by a growing body of basic clinical research. Arterial stiffness increases the afterload on the left ventricle, which can lead to concentric remodeling, myocardial fibrosis, and diastolic dysfunction, all of which are cardinal features of HFpEF.^24^ In stiff arteries, the aortic pulse pressure rises and reflected waves return earlier in systole, augmenting LV wall stress in late systole and impairing ventricular relaxation. Over time, this ventricular–arterial coupling mismatch elevates LV filling pressures and pulmonary congestion, which are central to HFpEF. Patients with HFpEF exhibited significantly greater arterial stiffness than did those without HFpEF.^25^ Basic science studies provide mechanistic insight: arterial stiffening is driven by inflammatory and fibrotic changes in the vasculature (eg, collagen deposition, elastin fragmentation, and smooth muscle hypertrophy).^26^ These processes, along with chronic inflammation, oxidative stress, and endothelial dysfunction, are involved in the pathogenesis of HFpEF, suggesting a shared milieu linking vascular and myocardial stiffening.^27^ The high pulsatile load in stiff arteries can also cause microvascular damage in high-flow organs. In the heart, excessive pulsatility may contribute to coronary microvascular dysfunction and myocardial ischemia, exacerbating HFpEF symptoms.^28^ Thus, there is a strong plausibility that elevated arterial stiffness is not just a marker of risk but also a mediator in the development of HFpEF.

Our results are based on previous epidemiological studies that examined arterial stiffness and incident HF. The Framingham Heart Study and other studies were the first to establish that increased aortic stiffness (assessed by PWV) is correlated with a higher long-term risk of HF and cardiovascular events.^29^ In the Health ABC cohort of older adults, a higher carotid-femoral PWV was associated with a greater HF incidence in age- and blood pressure-adjusted models, although the significance was attenuated after full risk factor adjustment.^30^ This suggests that arterial stiffness, hypertension, and aging are closely associated. In contrast, in our study, a high CAVI remained predictive, independent of blood pressure and other factors, highlighting that CAVI, a blood pressure-independent stiffness index, captures risk beyond that conveyed by brachial blood pressure alone. These findings are consistent with those of previous CAVI-focused studies. A prospective study in Japan reported that a CAVI >9.5 (vs <7.5) was associated with a significantly elevated risk of all-cause mortality and HF events.^31^ Moreover, increased CAVI has been linked to subclinical cardiac structural changes such as higher LV mass and subtle systolic dysfunction on echocardiography.^7^, ^8^, ^9^ Carotid-femoral PWV is pressure-dependent, technically demanding, and less practical for large-scale clinical application. CAVI has the advantage of being relatively pressure-independent and easily obtained in routine practice. Our findings suggest that CAVI provides prognostic information comparable to that of carotid-femoral PWV, but head-to-head studies directly comparing these indices in predicting HF outcomes are warranted.

Our results suggest that CAVI may be a practical tool for early HF risk and cardiovascular death stratification. The ability of CAVI to modestly improve reclassification beyond conventional factors indicates that it may help identify individuals with subclinical vascular dysfunction who are at a higher risk of progression to HF. Notably, arterial stiffness is not currently included in most HF risk scores or guidelines. Although our findings raise the possibility that incorporating CAVI could refine risk stratification, particularly for HFpEF, further validation in larger and higher-risk populations is required before its use can be recommended in routine clinical assessments or preventive strategies.

The absolute risk reduction observed in this study was small given the low incidence of HF hospitalizations (≈1%). However, this finding should be interpreted in the context of the study population, which primarily consisted of individuals with cardiovascular risk factors but without advanced disease. In such relatively low-risk populations, even modest differences in risk may be clinically meaningful, particularly when applied to large numbers of individuals in primary prevention settings. Importantly, CAVI provided incremental prognostic information beyond established risk factors, suggesting its potential value as a risk stratification tool to identify subgroups at a higher risk who may benefit from closer monitoring or early intervention. Nevertheless, further validation in larger cohorts or populations at higher baseline risk is warranted to clarify the clinical utility of CAVI in predicting HF events.

Although our findings suggest there may be the prognostic value of CAVI, several limitations of this biomarker should be acknowledged. First, CAVI reflects global arterial stiffness from the aorta to the ankle and is influenced by both central and peripheral arterial properties, which may dilute its specificity for left ventricular afterload compared with central indices.^12^ Second, CAVI measurement requires a dedicated device and standardized conditions, and ethnic variability has not been fully established.^32^^,^^33^ Third, the existing literature on CAVI and HF prognosis remains limited, consisting primarily of small, single-center studies with heterogeneous populations, and our study is among the first multicenter prospective investigations. Consequently, replication in larger and more diverse cohorts is essential before CAVI can be adopted broadly for risk stratification.

### Study limitations

This study had several limitations. First, it was a subanalysis of the CAVI-J study and was observational, which limits its ability to draw causal inferences. Second, HF-related hospitalizations were predefined as secondary outcomes, and the number of HF events was small. Similarly, the number of composite endpoints was modest. Although the study was adequately powered to detect meaningful associations, a larger sample or a dedicated HF-focused cohort would enhance the precision of the effect estimates. Third, echocardiographic assessment of LVEF was not available for all participants at baseline, making it unclear whether any observed ejection fraction reduction occurred after enrollment. Fourth, the study was exclusive to the Japanese population with cardiovascular risk factors, which may limit its generalizability to other ethnic or lower-risk populations. Fifth, CAVI was measured only at baseline, and longitudinal changes in arterial stiffness were not evaluated. Given the previous evidence that worsening arterial stiffness is associated with adverse cardiovascular outcomes, serial CAVI measurements could provide additional prognostic information. Sixth, important potential confounders, including natriuretic peptide levels and echocardiographic parameters, such as diastolic function or LV mass, were not included in the analysis and may have influenced the observed associations. We also acknowledge that residual confounding cannot be fully excluded, and the inability to apply propensity score adjustment due to the low event rate is a limitation of our analysis. Seventh, the use of ROC-derived cutoffs within the same data set should be interpreted cautiously, as it introduces a risk of optimism bias. Ideally, cut points should be derived and validated in separate cohorts; however, this was not feasible in the present study owing to the small number of events. Finally, the small number of events also raises the possibility of model overfitting. Although the primary models were adjusted only for age and sex, the incremental analyses included multiple covariates, which may have exceeded the modeling capacity of the data set. This limitation should be considered when interpreting the apparent improvements in model performance.

## Conclusions

Elevated CAVI was independently associated with an increased risk of HF-related hospitalizations and cardiovascular death and provided modest incremental prognostic information beyond conventional risk models. Given its noninvasive and reproducible nature, CAVI may serve as an adjunctive tool for risk stratification; however, its discriminative ability remained limited, and the clinical utility should be interpreted with caution.

Perspectives **COMPETENCY IN MEDICAL KNOWLEDGE:** This study demonstrated that CAVI is an independent predictor of HF-related hospitalizations and cardiovascular death in patients with cardiovascular risk factors. Employing CAVI in clinical practice may enhance early identification of subclinical vascular dysfunction and improve preventive strategies. These findings support the development of competencies in medical knowledge, patient care, and system-based practices.

**TRANSLATIONAL OUTLOOK:** Despite its promise, CAVI is not currently integrated into standard HF risk models or guidelines. Further prospective studies are required to determine whether CAVI-guided interventions reduce the incidence of HF. Broader validation and health system integration are essential for translating CAVI into routine cardiovascular risk assessments.

## Funding support and author disclosures

This study was supported by the Japan Vascular Disease Research Foundation (Tokyo, Japan). The authors have reported that they have no relationships relevant to the contents of this paper to disclose.
