# Supplementary material for: Association Between Cardio-Ankle Vascular Index and Heart Failure Outcomes: Insights From a Prospective Multicenter Cohort
Source: JACC Adv. 2025 Aug 29;4(12):102187. doi: 10.1016/j.jacadv.2025.102187 (PMC12793861; doi:10.1016/j.jacadv.2025.102187)
Supplement: Supplemental Data [file mmc1.docx]

**SUPPLEMENTAL APPENDIX**

**Association between Cardio-Ankle Vascular Index and Heart Failure Outcomes: Insights from a Prospective Multicenter Cohort**

- Study protocol of CAVI-J (Page 2-30)
- The Investigators and Institutions Involved in the CAVI‐J (Prospective Multicenter Study to Evaluate Usefulness of Cardio‐Ankle Vascular Index in Japan)
- Supplemental Figure 1 (Page 31)
- Supplemental Table 1 (Page 32)
- **Study protocol of CAVI-J**

**Protocol for evaluating the cardio–ankle vascular index to predict cardiovascular events in Japan: A prospective multicenter cohort study**

Brief tile: CAVI-J study

| **Study Title** | Protocol for evaluating the cardio–ankle vascular index to predict cardiovascular events in Japan: A prospective multicenter cohort study |
| --- | --- |
| **Protocol Date** | 01/April/2013 (Ver. 1.0)  27/March/2014 (Ver. 1.1) |
| **Study Chair** | Hajime Orimo, MD |
| **Funding** | The Japan Research Foundation for Healthy Aging |
| **Clinical and Data Coordinating Center** | 2-5-1 Shikata-cho, Okayama, Okayama University |
| **ClinicalTrials.gov Identifier** | NCT01859897 |

**TABLE OF CONTENTS**

1. Study Synopsis 3
2. Background and Rationale 4
3. Study Design 4
4. Study Objectives 4
5. Study Endpoints 5
6. Patient Population 5
7. Eligibility 5
8. Study Duration 8
9. Data Collection 8
10. Study-Specific Variables 9
11. Data Handling and Record Keeping 10
12. Study Organization 11
13. Quality Control and Assurance 12
14. Statistical Plan 14
15. References 14

Appendix: Definition of adverse events

1. **STUDY SYNOPSIS**

| **Title** | Protocol for evaluating the cardio–ankle vascular index to predict cardiovascular events in Japan: A prospective multicenter cohort study (CAVI-J) |
| --- | --- |
| **Coordinating Center** | 2-5-1 Shikata-cho, Okayama, Okayama University |
| **Study Chair** | Hajime Orimo, MD |
| **Overall Objective** | An open label, international, multicenter observational registry designed to examine the benefits of cardio–ankle vascular index (CAVI) as a predictor of cardiovascular events in high-risk patients. |
| **Study Design** | An open-label multicenter observational registry. The study will be conducted in up to 50 sites in Japan. |
| **Study Cohorts** | A total of 3,000 subjects undergoing CAVI will be enrolled. |
| **Eligibility** | **Inclusion Criteria:**   1. Adult individual between 40 and 74 years of age 2. Type 2 diabetes mellitus 3. Metabolic syndrome 4. Hypertension categorized as high-risk 5. Chronic kidney disease (stage 3) 6. History of coronary artery disease or cerebral infarction   **Exclusion Criteria:**  1.Under 40 years of age or over 75 years of age  2.Ankle brachial index < 0.9  3.Chronic atrial fibrillation  4.Heart failure (NYHA class III or IV) or left ventricular dysfunction (EF below 40%)  5.Medical history of cancer and/or treatment for cancer within the last 5 years  6.Estimated glomerular filtration rate <30 ml/min/1.73m^2^  7.Chronic hemodialysis  8.Treatment with systemic steroids or immunosuppressants  9.Liver cirrhosis  10.History of PCI/CABG within 6months  11.Severe valvular stenosis or regurgitation  12.Determined as unsuitable for this study by a physician |
| **Duration of Study** | Accrual is expected to take 6 years. All subjects enrolled will be followed-up for 5 years. Total duration of the study will be 6 years. |
| **Primary Endpoint** | 1. Cardiovascular death  2. Nonfatal myocardial infarction  3. Nonfatal stroke |
| **Secondary Endpoint** | 1. All cause death  2. Angina pectoris with revascularization  3. New incidence of peripheral arterial disease (arteriosclerosis obliterans)  4. Aortic aneurysm  5. Aortic dissection  6. Heart failure with hospitalization  7. Deterioration in renal function (chronic dialysis or kidney transplantation) |
| **Sample size** | 3,000 subjects |

1. **BACKGROUND AND RATIONALE**

Atherosclerosis is a major contributor to the development of cardiovascular diseases and thus a major cause of mortality and morbidity [1]. Reflecting the aging of society and adoption of westernized lifestyles, the number of patients with cardiovascular diseases is also increasing [2]. Risk factors for cardiovascular disease consist of male sex, advanced age, hypertension, diabetes mellitus, dyslipidemia, obesity, and smoking. Patients often have several risk factors [3]; these need to be carefully managed to prevent future cardiovascular events. The availability of a simple and noninvasive indicator for monitoring would be a powerful tool for managing atherosclerotic risk factors.

The cardio-ankle vascular index (CAVI) was developed in Japan and is a blood pressure-independent index of arterial stiffness from the origin of the aorta to the ankle [4]. In recent years, it has been studied by many researchers worldwide and it is strongly anticipated that it will play a role as a predictive factor for arteriosclerotic diseases. Published studies have shown that CAVI increases in the presence of cerebrovascular disease [5], dementia [6], cardiovascular disease [7-9], nephrosclerosis [10], vasculitis [11, 12], hypertension [13], hyperlipidemia [10], and lifestyle-related diseases including diabetes mellitus [14], smoking [15], sleep apnea syndrome [16], stress [17] and obesity [18], all of which are considered risk factors for arteriosclerosis. Recently, a single center study reported a positive association between high CAVI values and incidence of cardiovascular diseases [19]. However, no long-term multicenter prospective studies of this association have yet been reported.

1. **STUDY DESIGN**

CAVI-J is a prospective multicenter cohort study with central registration in Japan. The targeted population is heterogeneous, given the clinical use of CAVI across a number of indications. As such, patients referred for clinically indicated CAVI and meet the inclusion and the exclusion criteria will include those undergoing evaluation for atherosclerotic diseases. The study is considered non-significant risk because: 1) CAVI is a non- invasive diagnostic modality; 2) this is an observational registry with no targeted downstream alteration to the clinical care pathway of the patient or additional interventions. Up to 50 medical centers from Japan will participate in the study, 3,000 subjects will be enrolled into the study. Each Center may not enroll more than 20% of the total number of subjects.

1. **STUDY OBJECTIVES**

**Primary Objective.** To examine the benefits of CAVI as a predictor of primary endpoints (cardiovascular death, nonfatal myocardial infarction, and non-fatal stroke).

**Secondary Objectives.** The secondary objectives of CAVI-J include:

1. To determine the clinical implication of CAVI in each cardiovascular event, including

a) Primary endpoints: cardiovascular death, nonfatal myocardial infarction, and nonfatal stroke

b) Secondary endpoints: all cause death, angina pectoris with revascularization, new incidence of peripheral arterial disease (arteriosclerosis obliterans), aortic aneurysm, aortic dissection, heart failure with hospitalization, Deterioration in renal function (dialysis or renal transplantation)

2) To determine the clinical implication of CAVI in different patient subgroups, including:

a) Type 2 diabetes mellitus

b) Metabolic syndrome

c) Hypertension categorized as highest-risk

d) Chronic kidney disease (stage 3)

e) History of coronary artery disease or cerebral infarction

f) Sex

g) Age

h) Physical activity

i) Alcohol consumption

j) Medication at baseline

3) To examine the association of change in CAVI over time and cardiovascular events

1. **STUDY ENDPOINTS**

**Primary Endpoint (**Definition of each event is defined in Appendix file.)

1. Cardiovascular death

2. Nonfatal myocardial infarction

3. Nonfatal stroke

**Secondary Endpoint (**Definition of each event is defined in Appendix file.)

1. All cause death

2. Angina pectoris with revascularization

3. New incidence of peripheral arterial disease (arteriosclerosis obliterans)

4. Aortic aneurysm

5. Aortic dissection

6. Heart failure with hospitalization

7. Deterioration in renal function (dialysis or renal kidney transplantation)

# PATIENT POPULATION

# This study will prospectively enrol 3,000 patients undergoing CAVI. Sites participating in the CAVI-J of the study will be selected based on data quality and quantity of CAVI.

1. **ELIGIBILITY**

**Patient Eligibility and Screening.**

Patients who have presented or are presenting at a clinic or a hospital for clinical indication — and who meet the inclusion criteria and none of the exclusion criteria — will be included into CAVI-J study.

## Enrollment.

3,000 subjects will be enrolled, with no more than 20% of the total study population enrolled per site. Consecutive consenting adult patients who meet the inclusion criteria and none of the exclusion criteria will be asked to participate in the study.

## Ethical Considerations.

The study will be approved by the ethics committees of all hospitals involved. All participants provided written informed consent before enrollment. This study is conducted according to the principles expressed in the Declaration of Helsinki and is registered at ClinicalTrials.gov (NCT01859897).

**Informed Consent**

Study-specific data collection cannot be started until the patient has met all clinical inclusion criteria and written informed consent has been obtained. The investigator, or a person designated by the investigator who has been trained on the Investigational Plan, will explain the nature and scope of the study, potential risks and benefits of participation, and answer questions from the patient. If the patient agrees to participate, the informed consent form must be signed and personally dated prior to enrollment by the patient or his/her legally authorized representative and the investigator or a person designated by the investigator. A copy of the fully executed informed consent form must be provided to the patient. All patients must provide written informed consent in accordance with the ethics committee.

**Inclusion Criteria:**

patients between 40 and 74 years of age who have at least one of the following

1.Type 2 diabetes mellitus

2.Metabolic syndrome

3.Hypertension categorized as high-risk ^c)^

4.Chronic kidney disease (stage 3) ^d)^

5.History of coronary artery disease or cerebral infarction ^e)^

**Exclusion Criteria:**

1.Under 40 years of age or over 75 years of age

2.Ankle brachial index < 0.9

3.Chronic atrial fibrillation

4.Heart failure (NYHA class III or IV) or left ventricular dysfunction (EF below 40%)

5.Medical history of cancer and/or treatment for cancer within the last 5 years

6.Estimated glomerular filtration rate <30 ml/min/1.73m^2^

7.Chronic hemodialysis

8.Treatment with systemic steroids or immunosuppressants

9.Liver cirrhosis

10.History of PCI/CABG within 6months

11.Severe valvular stenosis or regurgitation

12.Determined as unsuitable for this study by a physician

**Definition of inclusion criteria**

**Type 2 diabetes mellitus:** Diabetes mellitus was defined according to the American Diabetes Association. [20]

**Metabolic syndrome:** Metabolic syndrome was defined with the modified criteria of the Japanese Expert Committee on the Diagnosis and Classification of Metabolic Syndrome for the clinical diagnosis of metabolic syndrome. [8] Waist circumference > 85cm (men) or > 90cm and at least two of following additional risks: fasting glucose > 110mg/dL, triglyceride > 150 mg/dL or HDL < 40mg/dL, and systolic blood pressure > 130mmHg or diastolic blood pressure > 85mmHg.

**Hypertension categorized as high-risk:** Hypertension categorized as high-risk was defined as a complication of diabetes mellitus or chronic kidney disease, or organ damages or multiple risk factors according to the guidelines of the Japanese Society for Hypertension 2009. [21]


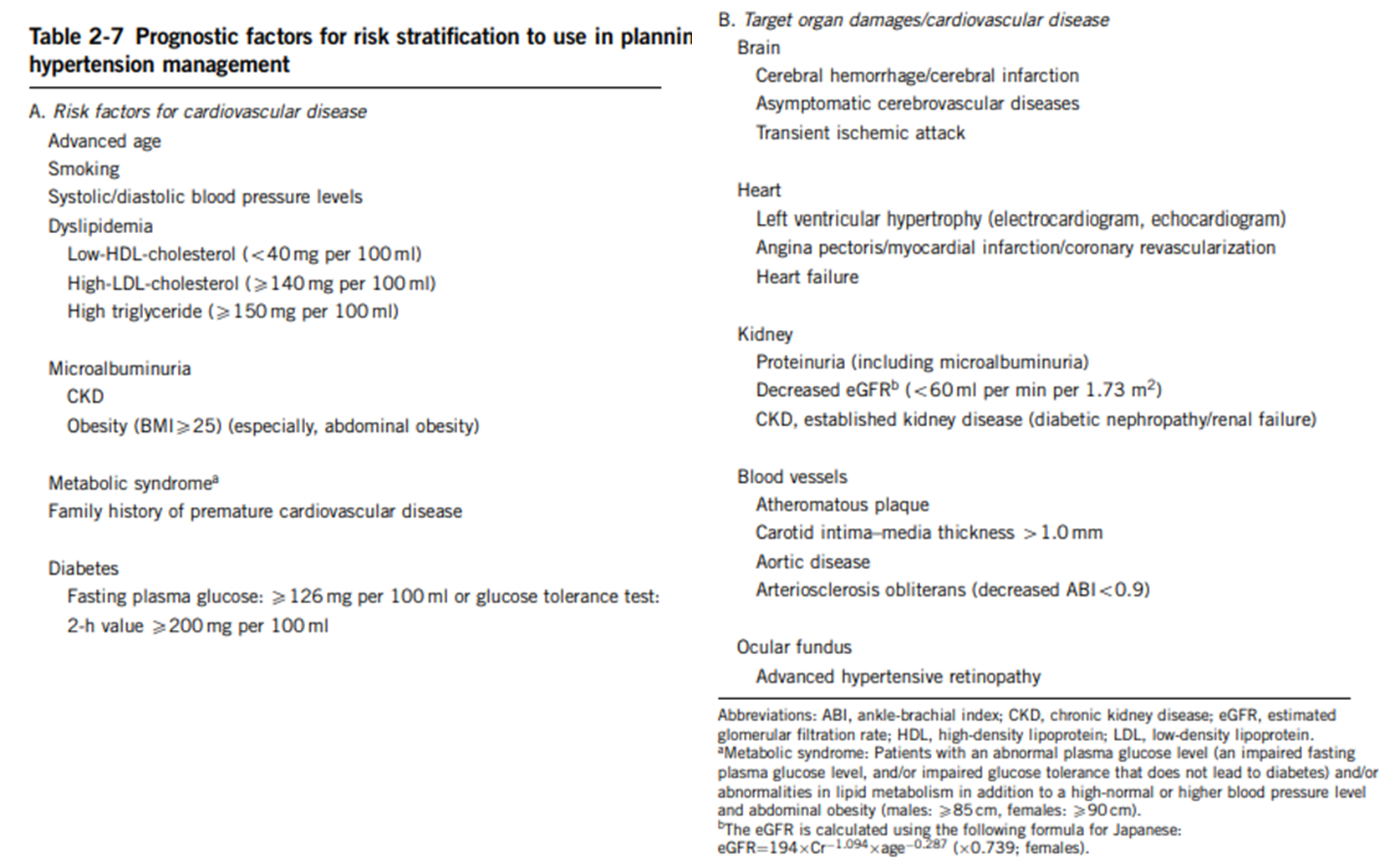


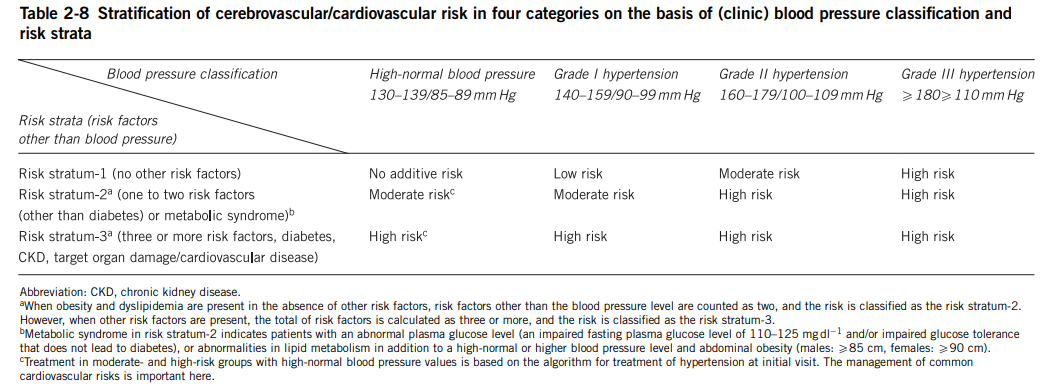


**Chronic kidney disease (stage 3):** Chronic kidney disease (stage 3) was defined as including patients with estimated glomerular filtration rates from 30 to 60 mL/min/1.73 m^2^ in accordance with clinical practice guidebook for diagnosis and treatment of chronic kidney disease 2012 [22]

**History of coronary artery disease or cerebral infarction:** History of coronary artery disease was defined as condition over 6 months after percutaneous coronary intervention or coronary artery bypass surgery. Coronary artery disease included angina pectoris, myocardial infarction, and unstable angina. Non-cardiogenic cerebral infarction is defined as cerebral infarction with an evidence by imaging modality (CT and MRI) except for cardioembolic infarction and intracerebral hemorrhage.

**Patient Discontinuation**

Every subject should remain in the study until completion of the required study period. However, a subject’s participation in any Clinical Investigation is voluntary and the subject has the right to withdraw at any time without penalty or loss of benefit.

**8. STUDY DURATION**

The anticipated duration of this study will be 6 years. Qualifying patients will be enrolled in the study for the first 1 years, and followed for an additional 5 years through follow up visits and/or phone calls.

| Activity | Start Day | Finish Day | Comments |
| --- | --- | --- | --- |
| **Site activation** | June 2013 |  |  |
| **Subject enrollment** | June 2013 | December 2014 |  |
| **Data monitoring** | June 2013 | May 2019 | Simultaneous with enrollment and follow up |
| **Data analysis** | December 2019 | --- |  |
| Abstracts / Publications | February 2020 | --- |  |

**9. DATA COLLECTION**

The following data will be collected:

|  | **Enrollment/Baseline** | **Year 1-5** |
| --- | --- | --- |
| Inclusion and Exclusion Criteria | **x** |  |
| CAVI | **x** | **x**** |
| Clinical Presentation | **x** |  |
| Demographics | **x** |  |
| CAD Risk Factors | **x** |  |
| Other Medical Conditions | **x** |  |
| Height and Weight | **x** |  |
| Laboratory Tests* | **x** |  |
| Medications | **x** |  |
| Drinking habit/physical activity |  |  |
| Primary and secondary outcomes | **x** | **x** |

* Laboratory test must be performed at same date of CAVI. ** Annual measurement of CAVI is not mandatory.

## 10. STUDY-SPECIFIC VARIABLES

Data dictionaries and case report forms will be provided to study investigators. These will include:

### Baseline Data and Laboratory Assessments

### Laboratory assessment and collection will be in accordance with standard hospital policy. In addition, the following assessments will be collected:

- Creatinine
- Total cholesterol
- HDL-C
- Triglycerides
- HbA1c
- Uric acid
- Urine protein (semi-quantitative)

Electrocardiogram

### Medical History and Clinical Presentation

**Demographics:**

- Age
- Sex
- Weight
- Height
- Weight circumflex
- Blood pressure
- Heart rate

**History of CAD:**

- Myocardial infarction
- Percutaneous coronary intervention
- Coronary artery bypass graft

**History of ischemic stroke**

**Concomitant medications:**

- Lipid-lowering agents – statins, ezetimibe, omega-3 fatty acid, etc.
- Antiplatelet therapy – aspirin, P2Y12-inhibitors
- Oral anti-coagulants – warfarin, DOACs
- Anti-hypertensive agents
- Diabetic agents – metformin, SGLT2 inhibitor, insulin, etc.
- Anti-osteoporosis agents

**CAD Risk Factors:**

- Diabetes mellitus
- Hypertension
- Dyslipidemia
- Smoking
- Atrial fibrillation
- Peripheral arterial disease
- Sleep apnea syndrome
- Chronic kidney disease
- Family history of diabetes, hypertension, and cardiovascular diseases

**Drinking habits:** how many times per week, how much volume per once converted as alcohol

**Physical activity:**

Moderate physical activity (>150 minutes per week), vigorous physical activity (> 75 minutes per week) or no physical activity (< 75 minutes per week) will be recorded [23].

**CAVI measurements:**

CAVI was measured using a CAVI device (Vasera; Fukuda Denshi, Tokyo, Japan). Electrocardiogram electrodes were placed on both wrists, a microphone for detecting heart sounds was placed on the sternum, and cuffs were applied to the upper arms and ankles bilaterally with the patient lying supine and the head held in the midline position. The examinations were performed after resting for 10 minutes. The pressure of all cuffs was kept low at 50 mmHg to minimize the effect of cuff pressure on hemodynamics. Blood pressure was then measured. Pulse wave velocity (PWV) was to be obtained by dividing vascular length by the time (T) taken for the pulse wave to travel from the aortic valve to the ankle. However, in practice T was difficult to obtain because the time the blood left the aortic valve was difficult to identify from the sound of the valve opening. Thus, because the time between the sound of the aortic valve closing and the notch of the brachial pulse wave is theoretically equal to the time between the sound of the aortic valve opening and the rise of the brachial pulse wave, T was obtained by adding the time between the sound of the aortic valve closing and the notch of the brachial pulse wave and the time between the rise of the brachial pulse wave and rise of the ankle pulse wave. CAVI was determined using the following formula: CAVI=a{(2ρ/∆P)×ln(Ps/Pd)PWV^2^}+b, where a and b are constants, ρ is blood density, ∆P is Ps–Pd, Ps is systolic blood pressure, and Pd is diastolic blood pressure.

# 11. DATA HANDLING AND RECORD KEEPING

The CAVI-J office will perform data management activities including documentation of the systems and procedures to be used. All e case report form (CRF) data collection will be performed through a secure web portal and all authorized personnel with access to the electronic data capture (EDC) system must use an electronic signature access method to enter, review or correct data. Passwords and electronic signatures will be strictly confidential.

The data will be subjected to consistency and validation checks within the EDC system. Completed eCRF images with the date-and-time stamped electronic audit trail indicating the user, the data entered, and any reason for change (if applicable) will be archived at the Investigator’s site and a backup copy archived with the CAVI-J office.

**Electronic Case Report Form Completion.**

Primary data collection based on source-documented hospital and/or clinic chart reviews will be performed clearly and accurately by the clinical site personnel trained on the protocol and eCRF completion. eCRF data will be collected for all patients that are registered into the study.

**Record Retention.**

The sponsor will archive and retain all documents pertaining to the study for the time of the study under evaluation, and for lifetime during the post-study phase. The Investigator must obtain permission from Executive Steering Committee (ESC) in writing before destroying or transferring control of any study investigation records.

**Publication Policy.**

All publications and other public disclosures related to the Study shall be by the decision of the ESC, in cooperation with the study investigators and clinical site. All publications or other disclosures must be approved in advance by the ESC.

Study investigators may use all study-related data for the purposes of scientific investigations, scientific abstracts, and scientific publications as has been approved by the ESC.

The CAVI-J office will be responsible for registering the study on clinicaltrials.gov, or any other clinical investigations, in accordance with the International Committee of Medical Journal Editors guidelines, or any other applicable guidelines.

# 12. STUDY ORGANIZATION

**Study Investigators.**

The Investigator(s) undertake(s) to perform CAVI-J in accordance with this protocol, ICH guidelines for Good Clinical Practice and the applicable regulatory local requirements.

The Investigator is required to ensure compliance with all procedures required by this protocol. The Investigator agrees to provide reliable data and all images into the EDC system in an accurate and timely fashion. The Investigator may appoint such other individuals as he/she may deem appropriate as Sub-Investigators to assist in the conduct of the study. All Sub-Investigators shall be appointed and listed. The Sub-Investigators will be supervised by and under the responsibility of the Investigator. The Investigator will provide them with a CAVI-J Protocol and all necessary information to successfully perform the study.

**Data Coordinating Center (DCC).**

As the DCC, CAVI-J office bears responsibility for monitoring interim data and analyzing the study's results in conjunction with the Investigators. Issues relating to regulatory reporting are the responsibility of both the Investigator and the DCC will aim to support these activities. The DCC will coordinate and monitor the study activities in alliance with the Principal Investigators, the ESC, and the sub-committees.

**Organizational and Leadership Design.**

The CAVI-J study organizational structure will comprise a study chair, principal investigators, an ESC; as well as dedicated disease-specific sub-committees.

**Study Chairs.**

The Study Chair will be responsible for the overall leadership of the study. The Study Chairs will work with site investigators and subcommittees, and will be responsible for reporting any pertinent findings to the ESC.

The Study Chair will be:

Hajime Orimo, MD

**Principal Investigators.**

Principal Investigators will be from investigative sites.

**Executive Steering Committee.**

The ESC is charge with the responsibility for ensuring scientific quality and study fairness. It is composed of the Study Chair, Principal Investigators, and Site Investigators. The ESC will meet at once a year to review study progress and conduct. The ESC will provide feedback to the CAVI-J office and study investigators after each meeting and on an *ad hoc* basis. In that capacity, The ESC will address and resolve scientific issues encountered during the course of the study. All final decisions regarding trial or protocol modifications rest with the ESC.

All proposed research investigations for CAVI-J study must be approved by the ESC.

The ESC membership will be comprised of the following (in alphabetical order):

1. Shigeo Horinaka, MD
2. Kohji Shirai, MD
3. Hiroshi Ito, MD
4. Jitsuo Higaki, MD

**Clinical Endpoint Review Committee**

The Clinical Endpoint Review Committee (CEC), consisting of members blinded to information about the patients, will assess the appropriateness of the clinical judgment of the cardiovascular events according to prespecified criteria.

The CEC membership will be comprised of the following:

1. Masanobu Takata, MD
2. Kuniaki Otsuka, MD
3. Shinichi Oikawa, MD

**Statistical consulting**

Shigeo Yamamura, PhD (Josai International University)

**13. Quality Control and Assurance**

**Site Qualification.**

Each clinical center will be required to obtain ethics committee approval for the protocol and consent (and their revisions) in a timely fashion, to recruit patients, to collect data and enter it accurately in the EDC system, to faithfully follow the protocol and adhere to the standards of Good Clinical Practice (GCP). Each participating site contributing patient-level data to CAVI-J study should meet the following site requirements:

- ability to organize data required for completion of CAVI-J case report form
- ability to perform de-identification of Protection Health Information (PHI) securely on-site in a manner in keeping with local regulations

**Investigator Profile.**

The following information will be collected for all investigators who participate in the study: CVs, contact information including address, telephone, and email, Conflict of Interest Statement and Financial Disclosure Certifications prior to initiation of enrollment.

**Qualifications and Training.**

Clinical investigators will be cardiology investigators with expertise in vascular function test including CAVI.

All clinical site investigators and coordinators will be trained by the DCC in the specifics of the protocol during site initiation in advance of patient enrollment. In addition, the investigators and coordinators will undergo a separate training session to gain familiarity with the EDC system.

**Safety Monitoring.**

Study Investigators and their site designees will be responsible for monitoring safety data throughout the course of the study.

**Delegation of Authority and PI Oversight.**

Principal Investigators are responsible for all study activities at their sites. They may delegate study tasks to qualified staff members while continuing to oversee all study activities. The Delegation of Authority Log will list each staff member’s title and responsibilities for the study. The PI is responsible for careful review of each staff member’s qualifications.

**Site Approval.**

The following documents must be collected prior to site approval and opening to patient enrollment:

- Signed Research and Data Use Study Agreement
- Signed Conflict of Interest Statements
- Completed Delegation of Authority Log
- Signed and dated CVs for all staff on Delegation of Authority Log
- Ethics committee approval for protocol, informed consent document
- Study-specific training documents
- Other regulatory and training documentation may be required prior to site initiation

Prior to enrolling a patient, representatives from the DCC will conduct a site initiation for all investigators, coordinators, and any other health care professionals who may be involved in the study.

**Patient Confidentiality.**

All patients’ records will be kept confidential. Study Investigators, CAVI-J representatives may review source documentation as necessary, but all unique patient and hospital identifiers will be removed from source documents which are sent to the CAVI-J office. The aggregate data from this study may be published as per publication policy documented in this Protocol; however, no data with patient identifiers will be published.

**14. Statistical Plan**

**Sample size**

Sample size calculations: The relative risk of cerebrovascular event in patients with CAVI>10 has been estimated to be 1.73 compared with patients with CAVI ≤10; thus, the study enrolled 2.5 times as many patients with CAVI ≤10 as patients with CAVI>10, [19] in whom the risk of cerebrovascular events is anticipated to be 4.6% in 5 years [24]. From these data, the risks of cerebrovascular events in patients with CAVI ≤10 and CAVI>10 were anticipated to be 0.038 and 0.066 in 5 years, respectively. To detect this risk difference, the required sample size was calculated by Freedman’s method to be 810 for CAVI ≤10 and 2024 for CAVI >10 groups with a two-sided alpha of 5%, 80% power and 20% dropout rate [25]. On the basis of these assumptions, a sample size of 3000 was chosen for this study.

**Analysis plan**

Data collection: categorical data will be presented as absolute numbers and percentages. Continuous data will be presented as mean ± standard deviation. Participants will be classified into several groups based on CAVI value. Baseline characteristics was compared among them. The effect of CAVI on each endpoint will be analyzed using the proportional hazard model. Incremental prognostic value was analyzed with likelihood ratio test, ROC (receiver operating characteristic) curve analysis, NRI (net reclassification improvement), and IDI (integrated discrimination improvement). The cutoff for CAVI against the incidence of cardiovascular events will be determined by ROC analysis.

**15. References**

[1] Roth GA, Forouzanfar MH, Moran AE, Barber R, Nguyen G, Feigin VL, et al. Demographic and epidemiologic drivers of global cardiovascular mortality. The New England journal of medicine. 2015;372:1333-41.

[2] Group NDR. Risk assessment chart for death from cardiovascular disease based on a 19-year follow-up study of a Japanese representative population. Circulation journal : official journal of the Japanese Circulation Society. 2006;70:1249-55.

[3] Yusuf S, Hawken S, Ounpuu S, Dans T, Avezum A, Lanas F, et al. Effect of potentially modifiable risk factors associated with myocardial infarction in 52 countries (the INTERHEART study): case-control study. Lancet (London, England). 2004;364:937-52.

[4] Shirai K, Utino J, Otsuka K, Takata M. A novel blood pressure-independent arterial wall stiffness parameter; cardio-ankle vascular index (CAVI). Journal of atherosclerosis and thrombosis. 2006;13:101-7.

[5] Suzuki J, Sakakibara R, Tomaru T, Tateno F, Kishi M, Ogawa E, et al. Stroke and cardio-ankle vascular stiffness index. Journal of stroke and cerebrovascular diseases : the official journal of National Stroke Association. 2013;22:171-5.

[6] Yamamoto N, Yamanaka G, Ishikawa M, Takasugi E, Murakami S, Yamanaka T, et al. Cardio-ankle vascular index as a predictor of cognitive impairment in community-dwelling elderly people: four-year follow-up. Dementia and geriatric cognitive disorders. 2009;28:153-8.

[7] Nakamura K, Tomaru T, Yamamura S, Miyashita Y, Shirai K, Noike H. Cardio-ankle vascular index is a candidate predictor of coronary atherosclerosis. Circulation journal : official journal of the Japanese Circulation Society. 2008;72:598-604.

[8] Matsuzaki Y et al. J Jpn Soc Int Med. 2005; 94:188–203(In Japanese).

[9] Horinaka S, Yabe A, Yagi H, Ishimura K, Hara H, Iemua T, et al. Comparison of atherosclerotic indicators between cardio ankle vascular index and brachial ankle pulse wave velocity. Angiology. 2009;60:468-76.

[10] Nakamura K, Iizuka T, Takahashi M, Shimizu K, Mikamo H, Nakagami T, et al. Association between cardio-ankle vascular index and serum cystatin C levels in patients with cardiovascular risk factor. Journal of atherosclerosis and thrombosis. 2009;16:371-9.

[11] Sato H, Miida T, Wada Y, Maruyama M, Murakami S, Hasegawa H, et al. Atherosclerosis is accelerated in patients with long-term well-controlled systemic lupus erythematosus (SLE). Clinica chimica acta; international journal of clinical chemistry. 2007;385:35-42.

[12] Masugata H, Senda S, Himoto T, Murao K, Dobashi H, Kitano Y, et al. Detection of increased arterial stiffness in a patient with early stage of large vessel vasculitis by measuring cardio-ankle vascular index. The Tohoku journal of experimental medicine. 2009;219:101-5.

[13] Miyashita Y, Saiki A, Endo K, Ban N, Yamaguchi T, Kawana H, et al. Effects of olmesartan, an angiotensin II receptor blocker, and amlodipine, a calcium channel blocker, on Cardio-Ankle Vascular Index (CAVI) in type 2 diabetic patients with hypertension. Journal of atherosclerosis and thrombosis. 2009;16:621-6.

[14] Ibata J, Sasaki H, Kakimoto T, Matsuno S, Nakatani M, Kobayashi M, et al. Cardio-ankle vascular index measures arterial wall stiffness independent of blood pressure. Diabetes research and clinical practice. 2008;80:265-70.

[15] Noike H, Nakamura K, Sugiyama Y, Iizuka T, Shimizu K, Takahashi M, et al. Changes in cardio-ankle vascular index in smoking cessation. Journal of atherosclerosis and thrombosis. 2010;17:517-25.

[16] Kumagai T, Kasai T, Kato M, Naito R, Maeno K, Kasagi S, et al. Establishment of the cardio-ankle vascular index in patients with obstructive sleep apnea. Chest. 2009;136:779-86.

[17] Himeno A, Satoh-Asahara N, Usui T, Wada H, Tochiya M, Kono S, et al. Salivary cortisol levels are associated with outcomes of weight reduction therapy in obese Japanese patients. Metabolism: clinical and experimental. 2012;61:255-61.

[18] Satoh N, Shimatsu A, Kato Y, Araki R, Koyama K, Okajima T, et al. Evaluation of the cardio-ankle vascular index, a new indicator of arterial stiffness independent of blood pressure, in obesity and metabolic syndrome. Hypertension research : official journal of the Japanese Society of Hypertension. 2008;31:1921-30.

[19] Kubota Y, Maebuchi D, Takei M, Inui Y, Sudo Y, Ikegami Y, et al. Cardio-Ankle Vascular Index is a predictor of cardiovascular events. Artery Research. 2011;5:91-6.

[20] American Diabetes Association: clinical practice recommendations 1999. Diabetes Care. 1999;22 Suppl 1:S1-114.

[21] Ogihara T, Kikuchi K, Matsuoka H, Fujita T, Higaki J, Horiuchi M, et al. The Japanese Society of Hypertension Guidelines for the Management of Hypertension (JSH 2009). Hypertension research : official journal of the Japanese Society of Hypertension. 2009;32:3-107.

[22] Japan nephrology s. [Special issue: Clinical practice guidebook for diagnosis and treatment of chronic kidney disease 2012]. Nihon Jinzo Gakkai Shi. 2012;54:1034-191.

[23] Goldstein LB, Whitsel LP, Meltzer N, Schoeberl M, Birnbaum J, Nelson S, et al. American Heart Association and nonprofit advocacy: past, present, and future. A policy recommendation from the American Heart Association. Circulation. 2011;123:816-32.

[24] Yamazaki T, Kohro T, Chujo M, Ishigaki M, Hashimoto T. The occurrence rate of cerebrovascular and cardiac events in patients receiving antihypertensive therapy from the post-marketing surveillance data for valsartan in Japan (J-VALID). Hypertension research : official journal of the Japanese Society of Hypertension. 2013;36:140-50.

[25] Freedman LS. Tables of the number of patients required in clinical trials using the logrank test. Stat Med. 1982;1:121-9.

- **Data S1. Definitions for adverse events**

**Appendix:** **Definition of adverse events**

| **Primary Endpoint** | 1. Cardiovascular death  2. Nonfatal myocardial infarction  3. Nonfatal stroke |
| --- | --- |
| **Secondary Endpoint** | 1. All cause death  2. Angina pectoris with revascularization  3. New incidence of peripheral arterial disease (arteriosclerosis obliterans)  4. Aortic aneurysm  5. Aortic dissection  6. Heart failure with hospitalization  7. Deterioration in renal function (dialysis or renal transplantation) |

**Cardiovascular death**

The cause of death will be determined by the principal condition that caused the death, not the immediate mode of death. CEC members will review all available information and use their clinical expertise to adjudicate the cause of death. All deaths not attributed to the categories of cardiovascular (CV) death and not attributed to a non-CV cause are presumed CV deaths and are part of the CV mortality outcome. Death certificates or summaries, if possible, including the date of death and other relevant details, will be provided for all patients who have died. However, if a death certificate is the only information available for review in addition to the patient data in the clinical trial database, the CEC may decide not to use this information as cause of death if another etiology appears more plausible. The following definitions will be used for the adjudication of fatal cases:

Sudden cardiac death. Death that occurs unexpectedly in a previously stable patient and includes the following:

• Witnessed and instantaneous death without new or worsening symptoms

• Witnessed death within 60 minutes of the onset of new or worsening cardiac symptoms

• Witnessed death attributed to an identified arrhythmia (e.g., captured by electrocardiogram or witnessed on a monitor by either a medic or paramedic)

• Subject unsuccessfully resuscitated from cardiac arrest or successfully resuscitated from cardiac arrest that dies within 24 hours without identification of a non-cardiac etiology

• Un-witnessed death with no conclusive evidence of another, non-CV, cause of death (i.e. presumed CV death).

Sudden death attributable to acute myocardial infarction (MI) (MI type 3). Sudden death occurring up to 14 days after a documented acute MI (verified either by the diagnostic criteria outlined for acute MI or by autopsy findings showing recent MI or recent coronary thrombus) where there is no conclusive evidence of another cause of death. If death occurs before the biochemical confirmation of myocardial necrosis can be obtained, adjudication should be based on clinical presentation and ECG evidence.

Death attributable to heart failure or cardiogenic shock. Death occurring in the context of clinically worsening symptoms and/or signs of congestive heart failure (CHF) without evidence of another cause of death.

New or worsening signs and/or symptoms of CHF include any of the following:

• New or increasing symptoms and/or signs of heart failure requiring the initiation of, or an increase in, treatment directed at heart failure or occurring in a patient already receiving maximal therapy for heart failure

• Heart failure symptoms or signs requiring continuous intravenous therapy or oxygen administration

• Confinement to bed predominantly because of heart failure symptoms

• Pulmonary edema sufficient to cause tachypnea and distress not occurring in the context of an acute MI or as the consequence of an arrhythmia occurring in the absence of worsening heart failure

• Cardiogenic shock not occurring in the context of an acute MI or as the consequence of an arrhythmia occurring in the absence of worsening heart failure

– Cardiogenic shock is defined as systolic blood pressure (SBP) <90 mmHg for more than 1 hour, ack of response to fluid resuscitation and/or heart rate correction, and judged to be secondary to cardiac dysfunction and associated with at least one of the following signs of hypoperfusion:

1. Cool, clammy skin
2. Oliguria (urine output <30 mL/hour)
3. Altered sensorium
4. Cardiac index <2.2 L/min/m^2^

Cardiogenic shock can also be defined in the presence of SBP ≥90 mmHg or for a time period <1 hour if the blood pressure measurement or time period is influenced by the presence of positive inotropic or vasopressor agents alone and/or with mechanical support <1 hour. The outcome of cardiogenic shock will be based on CEC assessment and must occur after randomization. Episodes of cardiogenic shock occurring before and continuing after randomization will not be part of the study outcome. This category will include sudden death occurring during an admission for worsening heart failure

Death attributable to stroke or cerebrovascular event. Death occurring up to 30 days after a stroke that is either attributable to the stroke or caused by a complication of the stroke.

Death attributable to other CV causes. Death must be caused by a fully documented CV event not included in the above categories (e.g. dysrhythmia, pulmonary embolism, or CV intervention). Death attributable to an MI that occurs as a direct consequence of a CV investigation/procedure/operation will be classified as death due to another CV cause.

Non-CV death

Non-CV death is defined as any death not covered by cardiac death or vascular death. The CEC will be asked to determine the most likely cause of non-CV death. Examples of non-CV death are pulmonary causes, renal causes, gastrointestinal causes, infection (including sepsis), non-infectious causes (e.g., systemic inflammatory response syndrome), malignancy (i.e., new malignancy, worsening of prior malignancy), hemorrhage (not intracranial), accidental/trauma, suicide, non-CV organ failure (e.g., hepatic failure) or non-CV surgery.

**Myocardial infarction (non-fatal)**

The term MI should be used when there is evidence of myocardial necrosis in a clinical setting consistent with myocardial ischemia. Under these conditions, any one of the following criteria (A and B) meets the diagnosis for myocardial infarction.

A. Spontaneous MI (type 1)

To identify a type 1 MI, patients should demonstrate spontaneous symptoms of myocardial ischemia unprovoked by supply/demand inequity, together with ≥1 of the following criteria:

• Cardiac biomarker elevation: Troponin is the preferred marker for adjudicating the presence of acute MI. At least one value should show a rise and/or fall from the lowest cut-point providing 10% imprecision (typically the upper reference limit for the troponin run per standard of clinical care). Creatine kinase-MB is a secondary choice of marker to troponin; a rise in CK-MB above the local upper reference limit would be consistent with myocardial injury.

• ECG changes consistent with new ischemic changes

– ECG changes indicative of new ischemia (new ST-T changes or new left bundle branch block [LBBB]) or ECG manifestations of acute myocardial ischemia (in the absence of left ventricular hypertrophy [LVH] and LBBB):

– Development of pathological Q waves in the ECG

1. Any Q-wave in leads V2–V3 ≥0.02 seconds or QS complex in leads V2 and V3
2. Q-wave ≥0.03 seconds and ≥0.1 mV deep or QS complex in leads I, II, aVL, aVF, or V4-V6 in any two leads of a contiguous lead grouping (I, aVL, V6; V4-V6; II, III, and aVF)

– ST elevation: New ST elevation at the J-point in two contiguous leads with the cut-off points: ≥0.2 mV in men or ≥0.15 mV in women in leads V2–V3 and/or ≥0.1 mV in other leads

– ST depression and T-wave changes: New horizontal or down-sloping ST depression ≥0.05 mV in two contiguous leads and/or T inversion ≥0.1 mV in two contiguous leads with prominent R-wave or R/S ratio >1

• Imaging evidence of new non-viable myocardium or new wall motion abnormality

B. “Demand”-related (type 2) MI

Patients with type 2 MI should be considered under similar diagnostic criteria as a type 1 MI; however, type 2 MI should be considered present when myocardial ischemia and infarction are consequent to supply/demand inequity, rather than a spontaneous plaque rupture and coronary thrombosis.

C. Percutaneous coronary intervention (PCI)-related MI (type 4a/4b)

For PCI in patients with normal baseline troponin values, elevations of cardiac biomarkers above the 99th percentile URL within 24 hours of the procedure are indicative of peri-procedural myocardial necrosis. By convention, increases of biomarkers >3 × 99th percentile URL (troponin or CK-MB >3 × 99th percentile URL) are consistent with PCI-related MI.

Where the cardiac biomarker is elevated prior to PCI, a ≥20% increase in the value of the second cardiac biomarker sample within 24 hours of PCI and documentation that cardiac biomarker values were decreasing (two samples ≥6 hours apart) prior to the suspected recurrent MI are consistent with PCI-related MI.

Symptoms of cardiac ischemia are not required.

D. Coronary artery bypass grafting (CABG)-related MI (type 5)

For CABG in patients with normal baseline troponin values, elevation of cardiac biomarkers above the 99th percentile URL within 72 hours of the procedure is indicative of peri-procedural myocardial necrosis. By convention, an increase of biomarkers >5 × 99th percentile URL (troponin or CK-MB >5 × 99th percentile URL) plus at least one of the following is consistent with CABG-related MI:

• New pathological Q waves in at least two contiguous leads on the ECG that persist for 30 days, or new LBBB

• Angiographically documented new graft or native coronary artery occlusion

• Imaging evidence of new loss of viable myocardium

If the cardiac biomarker is elevated prior to CABG, a ≥20% increase in the value of the second cardiac biomarker sample within 72 hours of CABG and documentation that cardiac biomarker values were decreasing (two samples ≥6 hours apart) prior to the suspected recurrent MI plus new pathological Q-waves in ≥2 contiguous leads on the electrocardiogram; or new LBBB, angiographically documented new graft, or native coronary artery occlusion; or imaging evidence of new loss of viable myocardium are consistent with a periprocedural MI after CABG. Symptoms of cardiac ischemia are not required.

Clinical classification of acute MI. Every MI identified by the CEC will be classified into one of the following categories:

• Type 1: Spontaneous MI related to ischemia arising from a primary coronary event such as plaque erosion and/or rupture, fissuring, or dissection

• Type 2: MI secondary to ischemia attributable to either increased oxygen demand or decreased supply, e.g. coronary artery spasm, coronary embolism, anemia, arrhythmias, hypertension, or hypotension

• Type 3: Sudden unexpected cardiac death, including cardiac arrest, often with symptoms suggestive of myocardial ischemia, accompanied by presumably new ST elevation, new LBBB, or evidence of fresh thrombus in a coronary artery by angiography and/or at autopsy, with death occurring before blood samples could be obtained or before the appearance of cardiac biomarkers in the blood

• Type 4a: MI associated with PCI

• Type 4b: MI associated with stent thrombosis as documented by angiography or at autopsy

• Type 5: MI associated with CABG

**Stroke (non-fatal)**

The rapid onset of a new persistent neurologic deficit attributed to an obstruction in cerebral blood flow and/or cerebral hemorrhage with no apparent non-vascular cause (e.g., trauma, tumor, or infection). Available neuroimaging studies must be considered to support the clinical impression and to determine if there is a demonstrable lesion compatible with an acute stroke. Strokes will be classified as ischemic, hemorrhagic, or unknown.

Diagnosis of stroke. For the diagnosis of stroke, the following four criteria should be fulfilled:

• Rapid onset of a focal/global neurological deficit with at least one of the following:

– Change in level of consciousness

– Hemiplegia

– Hemiparesis

– Numbness or sensory loss affecting one side of the body

– Dysphasia/aphasia

– Hemianopia (loss of half of the field of vision of one or both eyes)

– Other new neurological sign(s)/symptom(s) consistent with stroke

Note: If the mode of onset is uncertain, a diagnosis of stroke may be made provided that there is no plausible non-stroke cause for the clinical presentation

• Duration of a focal/global neurological deficit ≥24 hours OR <24 hours if attributable to at least one of the following therapeutic interventions:

– Pharmacologic (i.e., thrombolytic drug administration)

– Non-pharmacologic (i.e., neurointerventional procedure such as intracranial angioplasty)

or

– Available brain imaging clearly documents a new hemorrhage or infarct

or

– The neurological deficit results in death

• No other readily identifiable non-stroke cause for the clinical presentation (e.g., brain tumor, trauma, infection, hypoglycemia, peripheral lesion)

• Confirmation of the diagnosis by at least one of the following:*

– Neurology or neurosurgical specialist

– Brain imaging procedure (at least one of the followings):

1. CT scan
2. MRI scan
3. Cerebral vessel angiography

– Lumbar puncture (i.e. spinal fluid analysis diagnostic of intracranial hemorrhage)

If a stroke is reported but evidence of confirmation of the diagnosis by the methods outlined above is absent, the event will be discussed at a full CEC meeting. In such cases, the event may be adjudicated as a stroke on the basis of the clinical presentation alone, but full CEC consensus will be mandatory.

If the acute focal signs represent a worsening of a previous deficit, these signs must have either

• Persisted for more than one week

or

• Persisted for more than 24 hours and accompanied by an appropriate new CT or MRI finding

Classification of stroke. Strokes are sub-classified as follows:

• Ischemic (non-hemorrhagic): A stroke caused by an arterial obstruction attributable to either a thrombotic (e.g., large vessel disease/atherosclerotic or small vessel disease/lacunar) or embolic etiology. This category includes ischemic stroke with hemorrhagic transformation (i.e. no evidence of hemorrhage on an initial imaging study but appearance on a subsequent scan)

• Hemorrhagic: A stroke caused by a hemorrhage in the brain as documented by neuroimaging or autopsy. This category will include strokes attributable to primary intracerebral hemorrhage (intraparenchymal or intraventricular), subdural hematoma and primary subarachnoid hemorrhage

• Not assessable: The stroke type could not be determined by imaging or other means (e.g., lumbar puncture, neurosurgery, or autopsy) or no imaging was performed.

**Angina pectoris with coronary revascularization**

• Angina pectoris includes stable and unstable angina pectoris.

For diagnosis of unstable angina, the subject must first have had an episode of ischemic discomfort consistent with unstable angina (ischemic discomfort either at rest, of new onset, or in an accelerating pattern) lasting ≥10 minutes, which occurred before the subject presented to the hospital. However, if an increase of biomarkers >5 × 99th percentile URL (troponin or CK-MB >5 × 99th percentile URL) is observed, diagnosis will be myocardial infarction.

• Coronary Revascularization

Attempted revascularization procedures, even if not successful, will be counted. Revascularization is divided by type and urgency. Planned revascularization is defined as ischemia-driven coronary revascularization (PCI or CABG). The evaluation of invasive or non-invasive functional ischemia is essential before PCI. Urgent revascularization is defined as coronary revascularization (PCI or CABG) that occurred during a hospitalization prompted by unstable angina with an episode of ischemic discomfort at rest lasting at least 10 minutes.

**New incidence of peripheral arterial disease (arteriosclerosis obliterans)**

Diagnosis of PAD. Follow the below work flow of TASC II (J Vasc Surg. 2007 Jan;45 Suppl S:S5-67.)

The ankle–brachial index is a mandatory test.


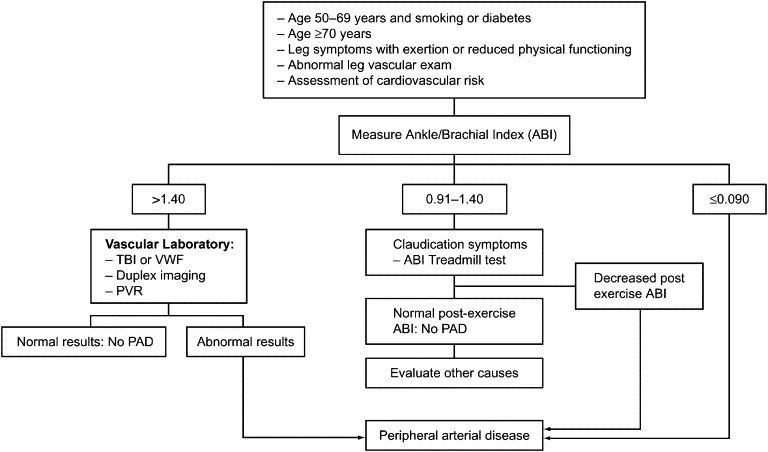


**Aortic aneurysm**

Aneurysm is defined as a segmental, full-thickness dilation of a blood vessel that is 50 percent greater than the normal aortic diameter (> 30 mm in abdominal aorta and >45 mm in thoracic aorta).

For diagnosis of aortic aneurysm, CT scan or MRI scan are mandatory.

**Aortic dissection**

Aortic dissection can result either from a tear in the intima and propagation of blood into the media or from intramural haemorrhage and haematoma formation in the media followed by perforation of intima; the former is more common. The characteristic picture of aortic dissection is the presence of an intimal flap in the aorta.

CT scan with contrast image enhancement is required to identify the extent of the dissection along with the true and false lumens

Classification of aortic dissection. DeBakey classification:

• Type I involves ascending aorta, aortic arch, and descending aorta.

• Type II is confined to ascending aorta only.

• Type III is confined to descending aorta distal to the left subclavian artery only; IIIa extends up to diaphragm, IIIb extends beyond the diaphragm.

**Heart failure with hospitalization**

The date of this event will be the day of hospitalization of the patient including any overnight stay at an emergency room or chest pain unit. Heart failure with hospitalization is defined as an event that meets all of the following criteria:

• Requires hospitalization defined as an admission to an inpatient unit or a visit to an emergency department that results in at least a 12-hour stay (or a date change if the time of admission/discharge is not available)

• Clinical manifestations of heart failure (new or worsening), including at least one of the followings:

– Dyspnea

– Orthopnea

– Paroxysmal nocturnal dyspnea

– Edema

– Pulmonary basilar crackles

– Jugular venous distension

– Third heart sound or gallop rhythm

– Radiological evidence of worsening heart failure

• Additional/increased therapy: at least one of the followings:

– Initiation of oral diuretic, intravenous diuretic, inotrope, or vasodilator therapy

– Up-titration of oral diuretic or intravenous therapy, if already on therapy

– Initiation of mechanical or surgical intervention (mechanical circulatory support, heart transplantation, or ventricular pacing to improve cardiac function); or the use of ultrafiltration, hemofiltration, or dialysis that is specifically directed at the treatment of heart failure

Changes in a biomarker (e.g., brain natriuretic peptide) consistent with CHF will support this diagnosis.

**Requirement for Renal Replacement Therapy**

Definition of renal replacement therapy:

• Kidney transplantation

Definitive renal replacement therapy prescribed when uremic symptoms have already occurred, or are anticipated to occur, due to the progression of irreversible chronic kidney disease. Death during the transplant surgery will be considered kidney transplantation.

• Chronic dialysis

ESKD will be diagnosed if dialysis is performed for 30 days or more and is not subsequently known to recover. Indications for dialysis are indicated in section below.

Onset of ESKD

The mode of onset of ESKD will be adjudicated into the following categories:

• Chronic progression

• Acute deterioration, diagnosed when the decline in kidney function is sudden and acute kidney injury is superimposed on chronic kidney disease resulting in renal replacement therapy.

**The Investigators and Institutions Involved in the CAVI‐J (Prospective Multicenter Study to Evaluate Usefulness of Cardio‐Ankle Vascular Index in Japan)**

Yuichi Akasaki (Kagoshima University Hospital), Noriko Asahara (Kyoto Medical Center), Masayuki Doi (Kagawa Prefectural Central Hospital), Tomikazu Fukuoka (Matsuyama Red Cross Hospital) Hiromichi Fukushima (Dokkyo Medical University), Yuji Hara (Hara Clinic), Koji Hasegawa (Kyoto Medical Center), Keiichi Hirano (Toho University Sakura Medical Center), Takashi Hitsumoto (Hitsumoto Medical Clinic), Toshio Honda (Sadamoto Hospital), Shigeo Horinaka (Dokkyo Medical University), Kotaro Ichinari (Hayato Onsen Hospital), Toshihiko Ishimitsu (Dokkyo Medical University), Kimihiko Ishimura (Dokkyo Medical University), Mai Iwataki (University of Occupational and Environmental Health), Hiroshi Kaieda (Taikai Clinic), Masahito Kajiya (Sumitomo Besshi Hospital), Shigeshi Kamikawa (Okayama Heart Clinic), Hitoshi Kaneko (Kaneko Clinic), Hideo Kawakami (Ehime Prefectural Imabari Hospital), Hajime Kihara (Kihara Cardiovascular Clinic), Yuko Kikuchi (Kyoto Medical Center), Hajime Kiyokawa (Toho University Sakura Medical Center), Takashi Kobayashi (Jyuzen General Hospital), Wataru Koguchi (Dokkyo Medical University), Mitsuteru Koizumi (Kyoto Medical Center), Kazuhiko Kotani (Jichi Medical University), Takuro Kubozono (Kagoshima University Hospital), So Kuwahata (Tarumizu Chuo Hospital), Motofumi Maguchi (Saijo Central Hospital), Mitsuru Masaki (Hyogo College of Medicine), Hitoshi Minowa (Minowa Naika), Michiaki Miyamoto (Aiseikai Clinic), Akihito Miyoshi (Tajiri Hospital), Kenichi Miyoshi (Ehime University Graduate School of Medicine), Toru Miyoshi (Okayama University Graduate School of Medicine), Maki Murata (Kyoto Medical Center), Mitsunobu Murata (Kokubunji Sakura Clinic), Tomoaki Nagao (Ehime University Graduate School of Medicine), Kazufumi Nakamura (Yura Hospital), Keigo Nakamura (Kagawa Prefectural Central Hospital), Michitsugu Nakamura (Saijo Central Hospital), Nobuyuki Nakano (Dokkyo Medical University), Seiji Nanba (Okayama Rosai Hospital), Kazuhisa Nishimura (Ehime University Graduate School of Medicine), Hachiro Obata (Okino Cardiovascular Hospital), Kazuro Ogurusu (Kasaoka City Hospital), Takefumi Oka (Tsuyama Chuo Hospital), Takafumi Okura (Ehime University Graduate School of Medicine), Madoka Onimaru (Onimaru Clinic), Shiro Ono (Saiseikai Yamaguchi General Hospital), Go Onoue (Onoue Clinic), Atsuhito Saiki (Toho University Sakura Medical Center), Satoru Sakuragi (Iwakuni Clinical Center), Toshihiro Sarashina (Tajiri Hospital), Koichi Seta (Kyoto Medical Center), Yoshimasa Shibata (Dokkyo Medical University), Kazuhiro Shimizu (Toho University Sakura Medical Center), Kohji Shirai (Mihama Hospital), Hiroyasu Sugiyama (Fukuyama City Hospital), Takumi Sumimoto (Kitaishikai Hospital), Sho Takahashi (Ibara City Hospital), Hitoshi Takehana (Sanseikai Clinic), Hiroshi Takeshima (Dokkyo Medical University), Masakatsu Todoroki (Dokkyo Medical University), Youkou Tominaga (Yashima General Hospital), Tadao Uraoka (Uraoka Clinic), Hiroshi Yagi (Dokkyo Medical University), Kensei Yahata (Kyoto Medical Center), Ryo Yoshioka (The Sakakibara Heart Institute of Okayama).

**Clinical Event Committee**

Masanobu Takata (Toyama Nishi General Hospital), Kuniaki Otsuka (Tokyo Women's Medical University), Shinichi Oikawa (Fukujuji Hospital and Nippon Medical School).

**Statistical Consulting**

Shigeo Yamamura, PhD (Josai International University).

**Figure S1 Competing Risk Analysis of Heart Failure-Related Hospitalization According to CAVI Tertiles**


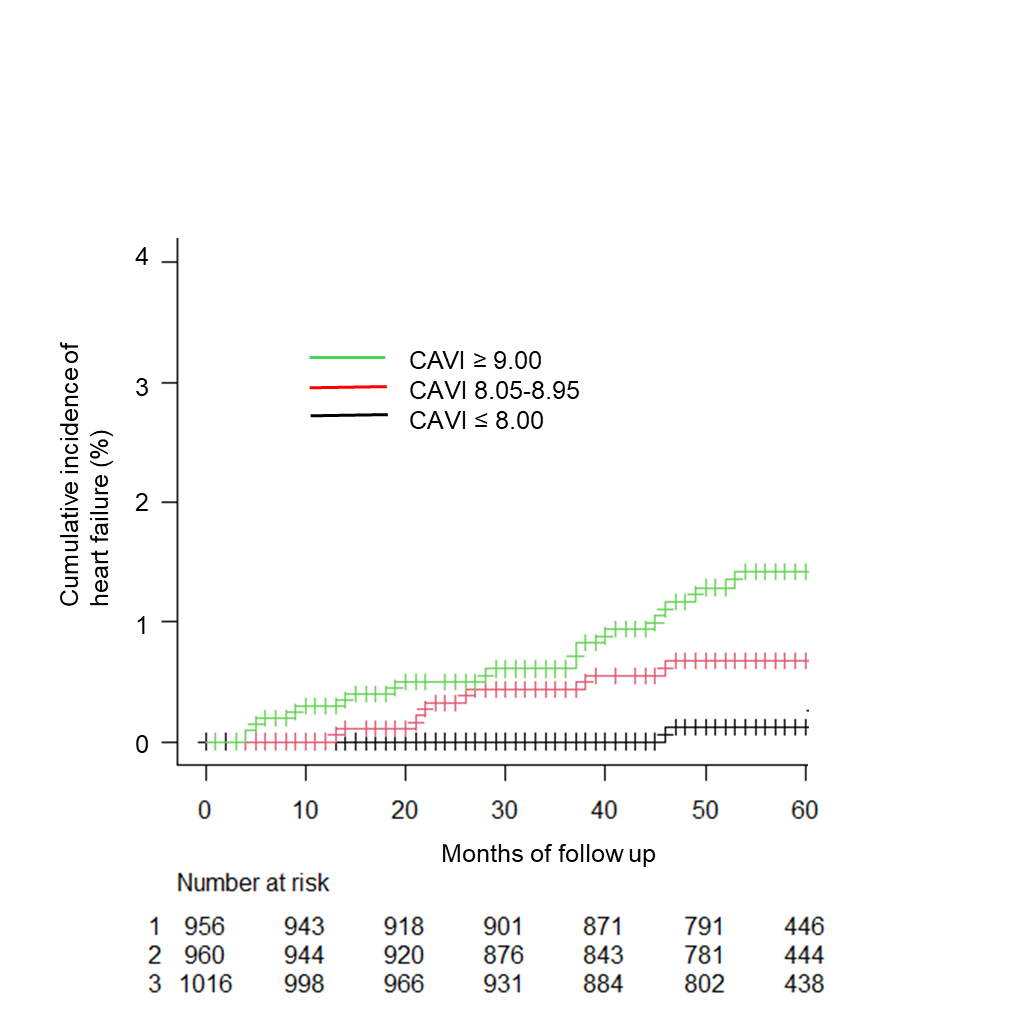


*P* < 0.001

Cumulative incidence function curves illustrating the risk of heart failure hospitalization stratified by CAVI tertiles, with cardiovascular death treated as a competing event. A significantly higher cumulative incidence was observed in the high CAVI group (T3) (Gray’s test *P* < 0.001).

**Table S1 Association of CAVI with heart failure-related hospitalization (Competing risk analysis)**

|  | Univariable | | |  | Multivariable* | | |
| --- | --- | --- | --- | --- | --- | --- | --- |
| CAVI | HR | 95%CI | P value |  | HR | 95%CI | P value |
| Primary outcome | |  |  |  |  |  |  |
| <8.0 | (reference) | | |  | (reference) | | |
| 8.0-9.0 | 3.041 | 0.615-15.03 | 0.170 |  | 3.100 | 0.682-14.130 | 0.140 |
| >9.0 | 6.289 | 1.427-27.71 | 0.015 |  | 6.436 | 1.472-28.380 | 0.013 |
| CAVI per 1 index | 1.591 | 1.188-2.131 | 0.002 |  | 1.573 | 1.156-2.141 | 0.004 |

*Adjusted for age, male sex
